# The role of immunotherapy sensitizers and novel immunotherapy modalities in the treatment of cancer

**DOI:** 10.3389/fonc.2024.1336546

**Published:** 2024-02-27

**Authors:** Guilherme Sacchi de Camargo Correia, Yujie Zhao, Rami Manochakian, Yanyan Lou

**Affiliations:** Division of Hematology and Medical Oncology, Mayo Clinic, Jacksonville, FL, United States

**Keywords:** immunotherapy, immune checkpoint inhibitors, immunotherapy sensitizer, tumor microenvironment, cancer immunology

## Abstract

The importance of the immune system in the response against cancer has always been a subject of intense investigation. The advent of immune checkpoint inhibitors has transformed the landscape of oncologic treatments, while expanding the understanding of this disease’s pathophysiology. Consequently, many therapies are being investigated, with interventions directed at different steps and pathways of the immune response. Relevantly, immunotherapy sensitizers have arisen as approaches focused on the synergistic effects of immunotherapy combination, or the combination of immunotherapy and other treatment modalities, such as chemotherapy or radiation therapy. Concomitantly, novel immunotherapy modalities are also in development. Approaches focusing from the tumor intrinsic pathways to the tumor microenvironment and ex-vivo interventions, such as CAR-T cell therapies and tumor-infiltrating lymphocytes are important examples. Although many of those interventions were initially envisioned as standalone options, their combination has demonstrated promising results in early-phase *in vitro* studies and clinical trials. The possibility of coupling different immunotherapy modalities, as well as with other techniques, further strengthen the concept of sensitizers, allowing for deeper and more robust responses in cancer treatment. This review aims to present an overview of the concepts of these sensitizing mechanisms that are the basis for the synergistic effects of immunotherapy combination, or the combination of immunotherapy and a multitude of therapeutic strategies. Novel immunotherapy modalities are also presented, focusing on the potential of combining them with sensitizer interventions. Understanding the complexity underlying these principles may be the key for future breakthroughs and improved patient outcomes.

## 1 Introduction

The potential roles of the immune system in treating neoplasia have been considered since the 19^th^ century. At that time, William B. Coley demonstrated the effects of the immune system in the treatment of bone and soft tissue sarcomas. This was performed by injecting bacteria and bacterial toxins into the tumor tissue (1).

From those first advancements, multiple physicians and scientists delved into this matter. Those important breakthroughs are beyond the scope of this review. However, important discoveries were made by James P. Allison and Tasuku Honjo. They established the roles of cytotoxic T lymphocyte-associated antigen 4 (CTLA-4) and programmed cell death protein 1 (PD-1) and their inhibition in cancer treatment (2–7). The relevance of their work was recognized with the 2018 Nobel Prize in Physiology or Medicine.

These scientific progresses led to clinical trials with drugs targeting CTLA-4 and PD-1. A trial of ipilimumab, a CTLA-4 inhibitor, demonstrated improved overall survival (OS) in patients with metastatic melanoma, leading to FDA approval of this drug in 2011 (8). Later, in 2014, pembrolizumab, a PD-1 inhibitor, was approved for the same patient population (9). Ultimately, targeting both pathways has also been shown to be effective (10).

Further progression in the field of immunotherapy led to numerous and significant approvals of immune checkpoint inhibitors (ICI) in diverse cancer types and stages. Indications now range from neoadjuvant and adjuvant settings to utilization for palliative intent in patients with metastatic disease. The importance of this class of drugs has also paved the way for the agnostic approval of pembrolizumab for tumors with deficient mismatch repair (dMMR) and high tumor mutational burden (TMB) (11).

However, despite its ubiquitous uses in oncology, there are still significant limitations to immunotherapy. The lack of uniform response in different diseases and different patients associated with mechanisms of resistance are some of the shortcomings observed in current practice (12). Despite having assumed a major role in the treatment of patients with melanoma, lung cancer, dMMR colorectal cancer, and Hodgkin lymphoma, the use of ICI has seen more limited use in diseases such as ovarian cancer and acute leukemias.

A relevant area in need of further development is the more accurate prediction of patients who will respond to immunotherapy. The most used markers in clinical practice include programmed cell death protein 1 ligand (PD-L1) expression, TMB, and MMR status. Other markers, that are being actively studied, include tumor-infiltrating lymphocytes (TILs), immune-associated gene expression, with many others still in development (13).

Nevertheless, associated with the investigations above, multiple ongoing research areas focus on either overcoming resistance to ICI or deepening and increasing the response to these agents. Significant developments have emerged in establishing potential new drug targets. Examples include lymphocyte-activation gene 3 (LAG-3), with its effectiveness again pioneered in patients with advanced melanoma (14), leading to FDA approval of the drug relatlimab in 2022. Among a wide spectrum of alternatives, encouraging targets are T-cell immunoreceptor with immunoglobulin and immunoreceptor tyrosine-based inhibition motif domain (TIGIT), TIM3 and B7H3. The diverse signaling pathways involving T and natural killer (NK) cells, dendritic cells, and regulatory T cells are an active area of research (15–20).

Furthermore, important advancements have focused on immunotherapy sensitizers. Sensitizers are interventions aimed at improving outcomes from immunotherapy. They can act synergistically with immunotherapy interventions, potentiating their effectiveness. But they can also participate in different immune system pathways with the objective of circumventing resistance mechanisms. Significantly, they may also be geared towards preventing or, minimally, delaying progression.

Immunotherapy sensitizer interventions include those directed at tumor cells, as well as the tumor microenvironment (TME). Pertinent new therapeutic targets and modalities have been discovered, ranging from targeting cell proteins and epigenetics to anti-cancer vaccines and oncolytic viruses. Importantly, the awareness about how other treatment procedures, such as classic chemotherapy or radiation therapy, interact with immunotherapy has also evidenced those as potential sensitizers.

Moreover, aside from upcoming clinical implications, these breakthroughs also shed light on the mechanisms involved in immunotherapy action and resistance. These insights allow for the development of additional sensitizing strategies and novel immunotherapy modalities. Chimeric antigen receptor T (CAR-T) cell therapy and the use of TILs are noteworthy examples of different strategies, while also being considered in association with other sensitizer methods.

In this review, we present some of the most significant immunotherapy sensitizer mechanisms currently in development or in early clinical investigation. Our goal is to present an overview of these mechanisms and upcoming therapeutic strategies focused on the relevant findings that are pertinent to the practicing medical oncologist. A detailed cell biology or pathophysiologic explanation of the targets for immune sensitization, or a thorough description of recently published or ongoing clinical trials are beyond the scope of this review.

## 2 Tumor-intrinsic pathways

Cancer response to immunotherapy relies on a complex pathophysiological background. The surrounding microenvironment and its interaction with the immune system play a major role, which will be addressed further in this review. Nonetheless, tumor cells themselves also participate in this process. Through the expression of antigens and intracellular and surface proteins, cells directly impact their interaction with the immune system.

One example is the β-catenin-dependent Wnt signaling pathway. This pathway extends from cell surface signaling to DNA transcription, leading to cell proliferation and resistance to regulatory mechanisms. In this way, it actively participates in carcinogenesis. However, the overactivation of this signaling pathway also correlates with decreased T-cell infiltration in tumors. It attenuates tumoral immune response through various mechanisms, including Foxp3 expression and regulatory T cells (Treg) proliferation (21).

In clinical practice, there has been a correlation between CTNNB1 mutations, as this gene encodes β-catenin, and resistance to ICI treatment in different cancers (22, 23). This association may indicate a potential utilization of CTNNB1 mutations as biomarkers to predict response to therapy (24). This mechanism lays the foundation for potentiating the effectiveness of immunotherapy in tumors with augmented Wnt signaling. Wnt inhibition with molecules such as OMP-18R5 and ETC-159 has led to enhanced efficacy of anti-PD-1 drugs in mouse models (25). Some early-phase clinical trials have also started to address this pathway with novel agents, including its utilization in association with ICI (26, 27).

Another contributor to the response to immunotherapy is the ephrin-A receptor 2 (EPHA2). This is a tyrosine kinase receptor that has been shown to be involved in tumoral immune regulation. Increased expression of this receptor has been correlated with decreased CD8 T-cell tumor infiltration. This hinders anticancer activity of ICI (28). Through active targeting and inhibition of EPHA2, this refractoriness to immune treatments may be reversed. Immunotherapy itself may be the key, through vaccines or CAR-T cells, to targeting cells expressing this receptor (29).

Finally, among a diversity of relevant and potential targets, mouse double minute 2 homolog (MDM2) is also involved in immune system evasion by tumors. MDM2 induces p53 degradation, which leads to decreased tumor-suppressor activity and, consequently, carcinogenesis (30). Besides this activity, overexpression of this protein provokes negative regulation of T-cells, decreasing immune activation. This may be another biomarker predicting a lack of response to ICI (31). The molecule AMG-232 is a MDM2 inhibitor that has been shown to sensitize MDM2-expressing cells to T-cell mediated killing (32). Phase 1 clinical trials have studied this compound as a single agent or in combination with other drugs in hematologic malignancies, demonstrating good tolerability (33, 34). Further investigations, including in different tumor types and potential associations with immunotherapy drugs, may exhibit eventual clinical uses in the future.

**Figure 1:**
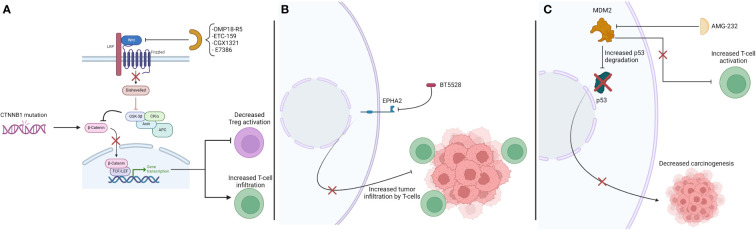
Interaction between therapies acting on tumor-intrinsic pathways and the immune system, leading to increased immune activation. **(A)** Drugs targeting CTNNB1 mutation and the Wnt pathway. **(B)** Drug targeting EPHA 2. **(C)** Drug targeting MDM2.

Tumor-intrinsic pathways are often associated with genetic mutations that translate into protein expression directly associated with decreased immune response. This may be a result of the increased activity of Treg, decreased CD8 activity, a combination of multiple mechanisms, among others. A summary of potential sensitizers presented in this section are listed on **Table 1**. The interaction between these drugs and the immune system is presented on **Figure 1**.

**Table 1:** Sensitizers focused on tumor-intrinsic pathways.

Investigational drug	Target	Immune system interaction
OMP18-R5 (25)	CTNNB1 mutation and Wnt pathway	CTNNB1 mutation leading to increased β-catenin activity. Increased β-catenin activity leads to overactivation of the Wnt pathway. Overactivation of the Wnt pathway causes decreased T-cell tumoral infiltration through Foxp3 expression and Treg proliferation.
ETC-159 (25)		
CGX1321 (26)		
E7386 (27)		
BT5528 (29)	EPHA2	EPHA2 increased expression leads to decreased tumor infiltration by CD8 T-cells.
CAR-T cell (29)		
Vaccine (29)		
AMG-232 (32–34)	MDM2	MDM2 amplification induces p53 degradation. Decreased p53 decreases tumor-suppressor activity, increasing carcinogenesis. MDM2 overactivation decreases T-cell activation.

## 3 Chemotherapy and radiation therapy-induced sensitization

Notwithstanding the importance and protagonist role assumed by immunotherapy and other novel anti-cancer therapies, classic chemotherapy, radiation therapy (RT), and surgical and other localized therapies are still central characters in the backbone of oncologic care. With new developments and better understanding of the molecular and cellular biology underlying anti-tumor response, the comprehension about the mechanisms of classic therapy lines has also expanded. With this expansion, the synergism between these interventions and newer treatments can expand the role and effectiveness of both, including immunotherapy efficacy.

Classic chemotherapy, especially cytotoxic agents, has the potential to stimulate immunologic response. There are many mechanisms underlying this event. An important and direct one is the enhancement of antigen and human leukocyte antigen class 1 (HLA1) expression in tumor cells (35). This mechanism increases antigen presentation, guiding the maturation of immune cells and their activity. Ultimately, cytotoxic chemotherapy can directly stimulate adaptive immune response, which represents a complex mechanism affecting dendritic cells, macrophages, and T cells (36).

However, antigenic stimulation is not the sole mechanism behind the immune-sensitizing effects of chemotherapy. Direct effects on the immune system can participate in its regulation and the anti-cancer response, mediated both by the adaptive, as mentioned above, and the innate immune response (37). The full array of mechanisms and interactions is extensive, but relevant examples include the effect of chemotherapy on Treg. Chemotherapy drugs, such as cyclophosphamide, can decrease their expression, alternatively stimulating immunologic response (38). A second example is gemcitabine, which decreases the numbers of myeloid suppressor cells, removing immune system inhibitors (39).

Similarly, RT also directly participates in recruiting anti-tumor immunologic activities (40). An example of this activity is the abscopal effect. It represents a systemic response leading to tumor regression in areas that are distant from the site undergoing radiation (41). Prior to the immunotherapy era, this effect was described as rare in the medical literature. However, the involvement of the immune system in this phenomenon has been considered the likely pathophysiological mechanism. Nonetheless, markers or predictors of this response are still unknown (42).

Once immunotherapy agents were adopted in clinical practice, this effect was once again observed. A study reported a case of a patient who presented the abscopal effect while receiving ipilimumab for metastatic melanoma (43). That study also shed light on the potential roles of the immune system, demonstrating changes in cancer-associated antigens prior to, with, and after radiation therapy at the time of disease response. Still, the occurrence of this effect is not widely observed, remaining as a disputable topic in clinical practice.

The underlying mechanisms for these effects triggered by RT are stipulated to be related to increased antigenic expression. Radiation induces double-stranded DNA breaks, causing cell death with cancer-associated antigens being released. It also leads to an increase in pro-inflammatory stimuli, such as interferon release. The combination of more antigens in a pro-inflammatory background leads to increased antigenic presentation, followed by a more robust immune response (44).

The immunogenic aftermath of RT is increased in the setting of immunotherapy. ICIs are thought to act both on naïve T-cells, promoting their expansion, but also through the reversal of T-cell exhaustion, especially CD8 positive cells (45). This synergism, with both therapies potentially stimulating the underlying inflammatory environment in the tumor, associated with more robust antigen presentation, is the basis for improved clinical outcomes with this combination.

**Figure 2:**
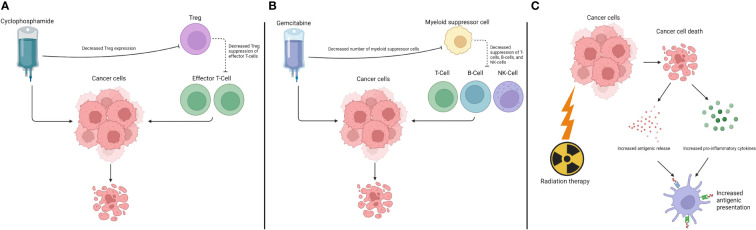
Interaction between chemotherapy drugs and radiation therapy and the immune system. **(A)** Effects of cyclophosphamide. **(B)** Effects of gemcitabine. **(C)** Effects of radiation therapy.

In conclusion, considering the complexity of cancer pathophysiology and the multiple mechanisms of resistance to therapy, targeting diverse pathways leads to synergy between interventions. Drugs and procedures that have been approved long before the first immunotherapy interventions still play a significant therapeutic role. The expansion of the understanding and utilization of immunotherapy will help determine immune system pathways activated by chemotherapy, radiation therapy, and other procedures. **Table 2** presents a brief outline of the synergistic mechanisms described in this text. **Figure 2** depicts the effects of chemotherapy agents and RT in the immune system.

**Table 2:** Sensitization from classic chemotherapy and radiation therapy.

Drug or therapeutic intervention	Mechanism of action	Immune system interaction
Cyclophosphamide (38)	Alkylating agent	Decreased Treg cells, ultimately stimulating the immune response.
Gemcitabine (39)	Pyrimidine antimetabolite	Decreased number of myeloid suppressor cells, with an overall diminished immune system inhibition.
Radiation therapy (43–45)	Double-stranded DNA breaks	Increased antigenic release from cancer cell death. Increased pro-inflammatory cytokines, such as interferon release. This association increases antigenic presentation.

## 4 Tumor-extrinsic pathways

Modulation of the anti-tumor immune response is complex and involves a multitude of pathways. Those that are focused outside of the tumor cells, or tumor-extrinsic, are directed towards the TME of cancer (46). This microenvironment is a rich biologic environment comprising immune cells, the extracellular matrix, microvascular and endothelial structures, and many other cell types and populations (47). Due to its complexity and intrinsic relationship with the primary tissue or organ involved by the disease, the TME differs based on the cancer type. Regardless of this variability, it plays a major role from tumor initiation to proliferation and, consequently, in the response to therapy. This is even more important in the response to immunotherapy.

T-cells are an essential component of the TME. One of the hallmarks of cancer, avoiding immune destruction, is directly tied to this cell population and the immune system (48). Tumors present mechanisms impairing the mobilization and migration of T and NK- cells, creating an immunosuppressive stimulus. This is paired with other mechanisms, such as decreased antigenic presentation (49). The assessment of different tumor types has led to further characterization and analysis of these effects on the TME, with the establishment of different categories of microenvironment immune penetration (50). Understanding these characteristics provides further insight into the pathophysiology of the disease, while also offering the substrate for therapeutic interventions, bolstering anti-tumor effects through more robust immune activation.

**Figure 3:**
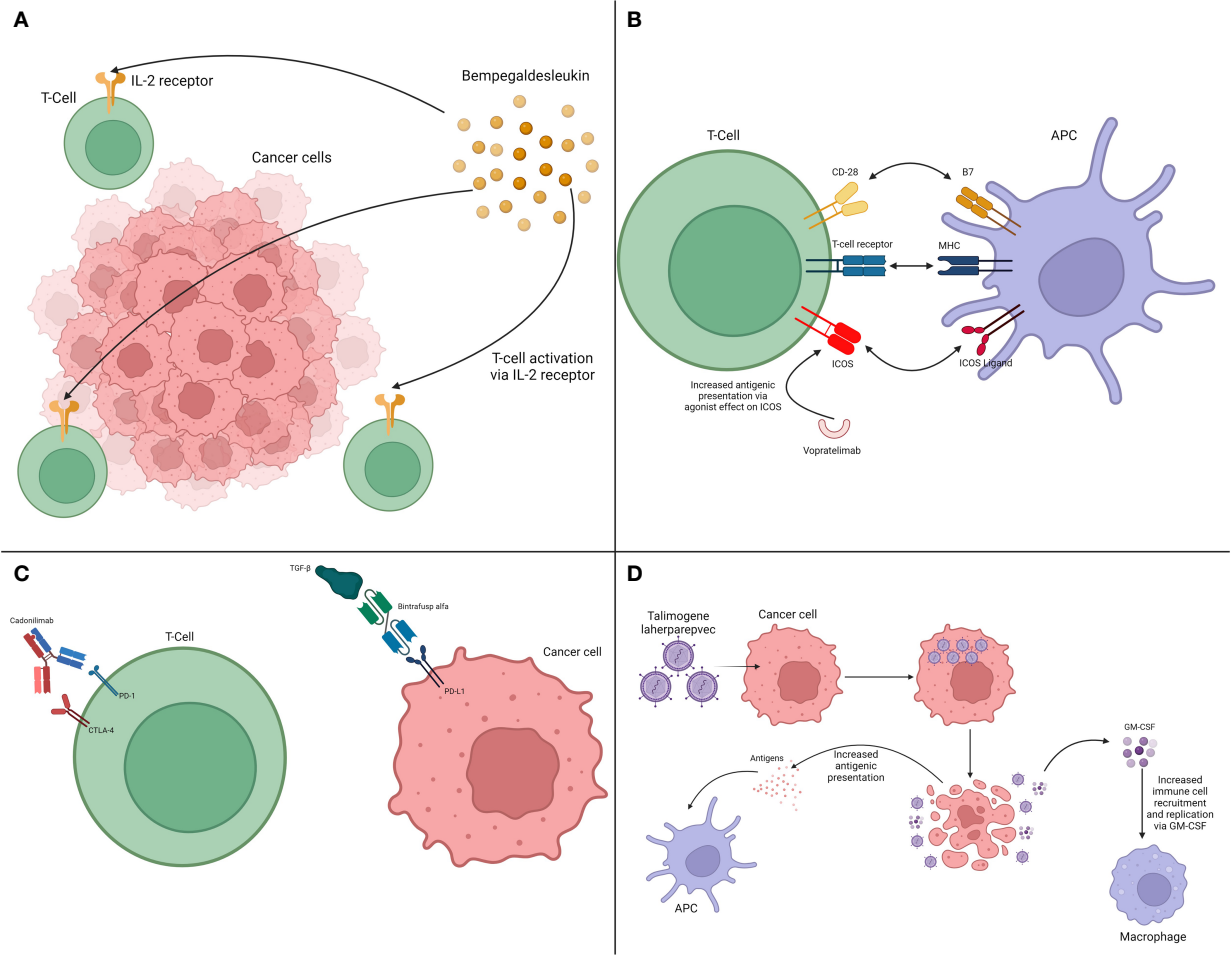
Interaction between therapies acting on tumor-extrinsic pathways and the immune system. **(A)** Cytokine modulation via IL-2 pathway. **(B)** Immune system activation via ICOS. **(C)** Bi-specific antibodies. **(D)** Oncolytic viruses.

The following sub-sections present strategies that are focused on the TME and other tumor-extrinsic pathways. In this way, bi-specific antibodies, vaccines, and oncolytic viruses are also discussed. The inclusion of these interventions is secondary to their ability to engage tumor-extrinsic pathways or cells reinforcing the immune response. The main methods presented below are summarized in **Table 3** and illustrated in **Figure 3**.

**Table 3:** Sensitizers focused on tumor-extrinsic pathways.

Investigational drug	Target or mechanism of action	Immune system interaction
Bempegaldesleukin (51, 52)	Pegylated IL-2 prodrug	T-cell and immune response activation via IL-2 pathway.
Vopratelimab (53)	ICOS agonist	Increased antigenic presentation and interaction between T-cells and tumor cells via CD28 superfamily.
Cadonilimab (54–56)	BiTE targeting PD-1 and CTLA-4	Checkpoint inhibition via blockade of signals from PD-1 and CTLA-4
Bintrafusp alfa (57–59)	BiTE targeting PD-1 and TGF-β	Checkpoint inhibition via blockade of signals from PD-1. Diminished immune evasion and blockade of pro-tumoral TME effects that originate from TGF- β.
Talimogene laherparepvec (60, 61)	Oncolytic herpes simplex virus type 1 with inserted GM-CSF gene	Increased antigenic presentation from tumor cell lysis. Increased immune cell recruitment and replication from GM-CSF activity.

### 4.1 Cytokine modulation

One direct way of supporting the anti-tumoral immune response is through cytokine stimulation. Due to their key role in the immune system, interleukin-2 (IL-2), for example, has been used to treat metastatic melanoma and renal cell carcinoma. However, despite receiving FDA approval considering positive results, relevant shortcomings were noticed, including significant toxicity (50). Despite that, research about the potential synergistic effect between cytokines, analogs, and ICI have occurred. One of these compounds is bempegaldesleukin, a pegylated IL-2 prodrug. It has been studied associated with the anti-PD-1 drug Nivolumab in patients with advanced melanoma. Unfortunately, the phase III clinical trial did not meet its primary endpoints, showing increased toxicity with the combination (51). Nevertheless, it is still under investigation in other diseases, such as sarcomas (52).

### 4.2 Immune system activators

Different strategies are investigating the immune activation pathways as potential targets that can be used in association with immunotherapy. A relevant target is the inducible T-cell costimulator (ICOS), a CD28-superfamily receptor (62). ICOS is present on the surface of T-cells, interacting with its ligand that is present on antigen presenting cells (APCs) leading to T-cell activation. Besides interacting with APCs, it also participates in the interaction between T-cells and tumor cells. Consequently, this has emerged as a fertile environment for immunotherapy sensitizing therapies (63). In this way, mouse tumor models have been used in the employment of bi-specific aptamers targeting both ICOS and tumor proteins, such as multidrug resistance protein 1. This strategy was combined with anti-CTLA-4 drugs, with results demonstrating promising antitumor response (64). First-in-human clinical trials have shown that the ICOS agonist vopratelimab to be safe, with an apparent correlation of efficacy in patients who harbor ICOS-high CD4 T-cells (53). Further studies are needed, but coupled with appropriate biomarkers, this strategy may become relevant in further clinical trials.

### 4.3 Bi-specific antibodies

Similarly to the concept of the bi-specific aptamers, the use of bi-specific antibodies is a prominent research concept being studied. Some of these molecules, also called bi-specific T-cell engagers (BiTE) have already been approved by the FDA, being widely adopted, especially in hematologic malignancies. One of the most relevant drugs in this class is blinatumomab, a BiTE that binds CD3 on the T cells and CD19 on the B-cell precursor blasts of acute lymphoblastic leukemia (ALL), that lead to prolonged overall survival in patients with ALL (65).

The basis of the mechanism of action of BiTE is their ability to target two different antigens, leading to their approximation. Once this occurs between a T-cell and a tumor cell, it can optimize the immune-effector response. Following this mechanism, BiTE can target an immune checkpoint receptor (e.g., PD-1, CTLA-4) and a stimulatory receptor (e.g., ICOS), target two immune checkpoint receptors, target a tumor antigen and an immune checkpoint receptor, or target an immune checkpoint receptor and cytokines (66). Outside of the realm of immune system directed therapies, other BiTE have been developed targeting surface proteins, such as amivantamab that has been approved for patients with non-small cell lung cancer harboring an epidermal growth factor receptor (EGFR) exon 20 insertion mutation (67).

One category of BiTE that is under investigation is the group of drugs targeting two immune checkpoint receptors. Those compounds follow the improved outcomes observed in some scenarios after the association of ICI targeting PD-1 and CTLA-4. A prime example of this concept is cadonilimab, a BiTE against PD-1 and CTLA-4. This was approved in China in 2022 for patients with recurrent or metastatic cervical cancer (54, 55), and being studied for other solid tumors (57). Importantly, another mechanism with promising expectations is targeting checkpoint receptors along cytokines. Drugs such as bintrafusp alfa, a BiTE directed against PD-L1 and transforming growth factor β (TGF-β), have been studied in different malignancies in phase I and II trials (57–59). Although results have not fully reached expectations, studies with this compound and drugs with similar concepts are ongoing, with encouraging prospects in the future. The developmental landscape of BiTE is vast. A comprehensive discussion is outside the scope of this review, but articles in the literature have summarized the main drugs and studies in the field (68).

### 4.4 Vaccines

The development of anti-cancer vaccine is another strategy that can direct and activate the immune system against cancer. Similarly to vaccine utilization in infectious diseases, a great diversity of techniques and mechanisms can be employed. These can be divided into two main categories according to the antigens being targeted. These can be predefined or not predefined. Predefined antigens can either be personalized, meaning they are patient-specific, or shared, representing cancer-specific antigens only that are common in an etiology. On the other hand, anti-cancer vaccines are considered not predefined when the immune cells are primed against tumor antigens that have not been previously selected. Vaccines in this class can be developed ex vivo, when APCs are collected and exposed to tumor antigens or can be utilized in situ. In this case the vaccine will direct the immune system to the tumor through viruses, APCs, bacteria, or receptor agonists. The immune recruitment is coupled by tumor-antigen release through other therapies (e.g., chemotherapy, radiation therapy) or tumor cell death (69).

Some vaccines have been already approved by the FDA, such as sipuleucel-T for patients with metastatic prostate cancer (70). However, their combination with other forms of immunotherapy, such as ICI, is a subject of investigation.

The coupling of both PD-1 and CTLA-4 inhibitors in association with GVAX, a vaccine that is composed by tumor cells modified to express granulocyte-macrophage colony-stimulating factor (GM-CSF) and irradiated to prevent their proliferation, showed increased effector response against tumor cells, with decreased Treg activity (71). This historical finding helped to pave the way for ongoing trials and strategies investigating this association.

Clinical trials have confirmed the sensitizing and synergistic activity of anti-cancer vaccines and ICI. Dendritic cell-based vaccines, when combined with Ipilimumab have demonstrated the potential of leading patients to durable complete remission (72).

Future perspectives in this field are encouraging. The more accessible implementation of molecular studies in oncology, associated with developing individualized and disease-specific antigenic panels, will help establish the foundation for further vaccine-focused work. Meanwhile, their association with immunotherapy will allow for more tailored immune system activation, with improved outcomes and, potentially, better tolerability (73, 74).

### 4.5 Oncolytic viruses

The concept of oncolytic viruses connects directly to the observations of William B. Coley and others, that infections and infectious organisms in tumors can promote positive cancer response. This concept was advanced and perfected with oncolytic viruses. These agents are structured to selectively invade and replicate in cancer cells, potentially leading to cell death and lysis, or in the delivery of specific particles into the tumor cells.

Oncolytic viruses have been approved by the FDA and utilized in clinical practice. One of the most significant ones is talimogene laherparepvec (T-VEC) in patients with unresectable stage III or IV melanoma (60). T-VEC is a genetically modified herpes simplex virus type 1 that is engineered to replicate within tumor cells, causing their lysis, and that is injected directly into target lesions. Importantly, the GM-CSF gene is inserted in its structure, resulting in increased localized factor production, recruiting the immune system to the affected area (61).

Diverse viral frameworks can be utilized for treatment purposes, with adenovirus being a frequent one. Despite that, a common pattern is their lytic potential. Through tumor lysis, they cause antigenic release in the tumor environment, potentially promoting an immunologic response. In this way, an association with ICI becomes more significant, as these drugs can assist in mitigating some of the regulatory or suppressive mechanisms that cancer exerts (75).

Although mechanistically interesting, a recent trial utilizing the already approved T-VEC associated with the PD-1 inhibitor pembrolizumab failed to meet its primary endpoints (76). Nonetheless, the combination was found to be safe. These results also confirmed that further mechanisms may play a role in the response to this treatment combination.

Finally, models are being established for other structures of oncolytic viruses. Through the potential of inserting genes in these agents leading to localized protein expression, cytokines and pro-inflammatory molecules can be generated through the viral invasion (77). This can be coupled with ICI, potentiating the response to both compounds. These encouraging breakthroughs are inspiring, demonstrating the potential of oncolytic viruses as immuno-modulating agents (78). Considering the other advancements that have been made in the field of immunotherapy, spearheaded by ICI, synergistic approaches will arise utilizing these strategies.

## 5 *Ex-vivo* immunotherapy interventions

The complexity of the immunologic response, exemplified by the plurality of parallel immune pathways, opens the doors for a vast diversity of strategies. Consequently, multiple strategies to engage and harvest the potentials from the immune system are being studied. Among those, ex-vivo interventions, revolving around collecting immune cells and modifying them, followed by re-infusion, are a relevant approach (79).

One of these strategies is the therapeutic use of TILs. The prognostic value of TILs is also important, with significant data supporting its importance in different malignancies. In breast and colorectal cancer, for example, the presence of higher levels of TILs in the tumor tissue is associated with longer OS (80, 81). Based on these findings, their therapeutic implications have been investigated. Their utilization consists of the extraction of TILs from tumor tissue. After this step, cells are expanded *in vitro* with the use of cytokines, such as IL-2. These cells may undergo a selection process, after which they are infused back into patients (82).

The immunogenic characteristics of melanoma allowed for the investigation of therapeutic TILs in this patient population (83). Initial trials demonstrated the potential of eliciting durable, complete responses with this intervention, which was later confirmed in a phase III clinical trial (84). This trial compared therapeutic TILs to ipilimumab in patients with advanced melanoma. The intervention arm demonstrated prolonged progression-free survival (PFS), objective response rates, and complete response (85).

However, considering the significant advances made with ICI, their relative ease of implementation, and the other potential breakthroughs in immunotherapy, questions arise regarding the incorporation of therapeutic TILs in this scenario. The combination of TILs and ICI is feasible and safe, potentially indicating a future step to be considered (86). In initial trials, the combination demonstrated superiority to ICI alone in PFS and OS in patients with metastatic osteosarcoma (87).

Another aspect to be considered regarding the eventual synergism between these two interventions is the sequencing and timing of the therapies. ICI *in vitro* in extracted TILs prior to their re-infusion has demonstrated increased reactivity of the lymphocytes, with better expansion of that cell population (88, 89). On the other hand, the addition of ICI after TILs infusion may potentiate the anti-tumor effect of those cells (90).

Another ex-vivo treatment approach is through CAR-T cells. These are genetically engineered T-cells to harbor a chimeric antigen receptor. This receptor, against a pre-specified tumor antigen, has a transmembrane domain with costimulatory fractions in its structure (91). CAR-T cells have been approved and widely used in hematologic malignancies, especially lymphomas (e.g., diffuse large B cell lymphoma, follicular lymphoma, mantle cell lymphoma) and multiple myeloma. Their long-term results confirmed their ability to induce disease remission, often long-lasting, and improved PFS compared to other therapeutic alternatives (92).

The utilization of CAR-T cells in other areas of oncology, such as solid tumors, presents important challenges. One of them is targeting a tumor antigen that is not otherwise expressed in normal tissue cells. Another barrier is the ability of these cells to penetrate solid tumors, leading to their expected effectiveness. Besides that, other regulatory stimuli, such as those in the tumor microenvironment, add another layer of complexity to this issue (93).

In this way, addressing these issues is relevant to the widespread adoption of CAR-T cells in other areas of oncology. Regardless of these potential blockades, however, sequencing of therapies is already a possibility. Due to both CAR-T cells and ICI acting on the immune system, questions may arise as to whether they may impair the immune system’s ability to respond to one or other interventions. Although trials have demonstrated some response derived from ICI use in patients whose disease progressed after CAR-T cell therapy, those cases were few and in select populations, with general outcomes still not reaching expectations (94). With a better understanding of the utilization of these therapies and their roles in different malignancies, better strategies for using them synergistically or sequentially will arise.

TILs are CAR-T cells are pivotal examples of immunotherapy strategies that are not ICI. Their development is more advanced when compared to some of the strategies discussed earlier in this review. Hence, some of these compounds have already been approved by regulatory agencies, being used in clinical practice. Analogously to interventions focused on tumor-intrinsic and -extrinsic pathways, they also deepen the understanding about tumor immunology. However, regardless of promising results, resistance mechanisms to CAR-T and TILs still arise, with cases of treatment failure as well. Accordingly, sensitization of these techniques, or their use as sensitizers of other drugs, for example may represent valid considerations to further broaden the effectiveness of anti-cancer treatment.

Importantly, newer therapies are not devoid of adverse events. Besides the short-term and immediate events following administration of these products, long-term events are now being reported more frequently. A relevant event associated with CAR-T cell therapy is the incidence of secondary malignancies (95). These malignancies can be either hematologic or solid tumors, raising significant concern that triggered a warning by the FDA in November 2023.

## 6 Discussion

Immunotherapy has become a more prevalent therapeutic strategy in oncology since the approval of the ICI ipilimumab by the FDA in 2011 (96). Following its approval in melanoma, a vast diversity of drugs and indications have seen light, significantly changing the treatment landscape of some diseases. However, although the term “immunotherapy” has been sometimes used as a synonym of ICI, other important forms of immunotherapy have either been approved or are undergoing constant investigation with promising results.

Among those other immunotherapy modalities, vaccines, oncolytic viruses, CAR-T cell therapy, and therapeutic TILs are some of the more prominent ones. Nonetheless, other essential advancements in terms of the safe and optimized utilization of cytokines, which have been used more commonly when newer compounds were not yet available, and in the diversity of molecules being targeted by BiTE, help shape the future of immunotherapy.

The relevance of this field spans beyond understanding the pathophysiology underlying cancer immune-evasion and the improved outcomes. It has also allowed the treatment of patient populations that otherwise would not tolerate traditional therapeutic modalities, such as classic chemotherapy. Although those populations, such as elderly and more frail patients, are underrepresented in clinical trials, immunotherapy efficacy in this group may approach what is observed in younger populations (97). Although further studies and the inclusion of these patients in upcoming trials are needed, these interventions, especially ICI, seem to be safe as well, with the caveat of presenting higher rates of treatment discontinuation due to toxicities (98).

Considering these advantages, further study of immunotherapy is encouraging. Importantly, not only the study of new modalities or drugs but the study of the combination of these agents may open further treatment opportunities. The complexity of the immune system and the immunologic response to cancer cannot be underscored. Consequently, addressing multiple of those pathways through sensitization mechanisms may represent ways to further improve outcomes and treatment tolerability of cancer patients.

Even so, the broad assortment of studies focusing on the immune system in cancer have illustrated the complexity of this system. A representative, but small, selection of negative studies and trials have been presented in this text. While providing a meaningful insight into how to further adapt and ameliorate interventions, negative results reinforce some of the challenges of this field. Some of these obstacles emerge from the translation of basic research models to human immunity. Others span from the complexity in correctly establishing the propelling pathways in cancer immunology. Similarly, there are tissue and organ-specific tumor factors that may impede the establishment of agnostic therapeutic strategies (99).

Meanwhile, clinical studies have an abundance of barriers in their development, interpretation, and betterment. Outcomes may be unpredictable, with some of this variability stemming from clinical and other variables, such as ethnicity, geographic, and cultural differences. The identification of reliable and consistent biomarkers also plays a significant role in the adequate applicability and effectiveness of new interventions. Late outcomes are impacted by long-term resistance mechanisms that may vary from a clinical to a laboratory setting (100).

Ultimately, practical constraints may pose challenges to promising advancements. Robust and late-phase clinical studies are needed prior to the approval of use of new agents in clinical practice. Finally, financial restraints may also hinder potential breakthroughs. The development of these new strategies is expensive and resource-demanding, which is also true once these strategies become applied in clinical studies and, later, in clinical practice.

Investigating ways to sensitize tumor cells to immunotherapy and to potentialize their effects has the potential to elicit more profound responses and longer survival times. Considering the abundant possibilities of using sensitizers and combining immunotherapy modalities, it is possible to trace a parallel with the combination of classic chemotherapy drugs. Multidrug regimens allowed for the targeting of multiple pathways within the tumor cells and in the cell cycle. Similarly, the combination of immunotherapy modalities may perform the same in terms of the immune system, the multitude of stimuli triggered by it, and the tumor microenvironment.

## Author contributions

GS: Conceptualization, Data curation, Investigation, Methodology, Writing – original draft, Writing – review & editing. YZ: Writing – review & editing. RM: Writing – review & editing. YL: Supervision, Writing – review & editing.
